# Knowledge and Attitudes towards HIV and HCV among the Population Attending the Fast-Track Cities Mobile Unit in Brescia, Italy

**DOI:** 10.3390/ijerph20196878

**Published:** 2023-10-03

**Authors:** Francesca Viola, Beatrice Formenti, Stefania Arsuffi, Itala Polesini, Emanuele Focà, Francesco Castelli, Eugenia Quiros-Roldan

**Affiliations:** 1 Division of Infectious and Tropical Diseases, University of Brescia and ASST Spedali Civili Hospital, Piazzale Spedali Civili, 25123 Brescia, Italy; ceccaviola@yahoo.it (F.V.);; 2 UNESCO Chair in Training and Empowering Human Resources for Health Development in Resource-Limited Countries, University of Brescia, Piazza del Mercato, 25121 Brescia, Italy

**Keywords:** HIV, HCV, health literacy, HIV knowledge, HCV knowledge, viral infections

## Abstract

The Infectious and Tropical Diseases Department of the University of Brescia organized free rapid screening tests for HIV and HCV as part of the Fast-Track City commitment. A cross-sectional study was conducted, consisting of an anonymous multiple-choice questionnaire that was administered to individuals who underwent the screening or consultation. The study aimed to compare knowledge and attitudes towards HIV and HCV between age groups (18–40 vs. >40) and sexual orientations (heterosexual vs. LGBTQ+). Overall, 333 questionnaires were completed. Overall, only 107 (32%) of respondents knew how HIV is transmitted. Major differences were shown between different age groups, where people under the age of 40 had a significantly higher correct response rate than people over 40 (n = 101; 39% versus n = 6; 7.8%, *p* < 0.00001). Similarly, almost half of LGBTQI+ people (n = 28; 44.4%) gave the correct answer, versus 30% (n = 79) of heterosexuals (*p* = 0.0359). Only 9.6% of the population demonstrated high levels of knowledge for both HIV and HCV. Our study highlights that misconceptions about HIV and HCV should be addressed in prevention and education programs, whose target should also be specific populations.

## 1. Background

Pursuing the global commitment of fighting HIV, viral hepatitis and tuberculosis (TB) has led to a partnership between international institutions and over 400 cities worldwide, signing the Paris Declaration on Fast-Track Cities in 2014. The Paris Declaration recognizes the urgency of these public health issues and sets goals for cities to accelerate their response to them [1].

Brescia, Northern Italy has one of the highest incidences of HIV (5.8/100,000 compared to 4.7/100,000 in Italy) [2]. This awareness highlights the need for increased efforts to address these epidemics. In becoming a Fast-Track City in 2020, Brescia has demonstrated the city’s commitment to address the issue and work towards achieving the fast-track goals.

The European Testing Week is a significant step towards achieving the fast-track goals [3] and this. The initiatives aim to offer Voluntary Counseling and Testing (VCT) for HIV and for viral hepatitis C (HCV) to the general population and to promote awareness on the benefits of the earlier HIV and hepatitis testing. Since 2013, the World Health Organization Regional Office for Europe has encouraged participation in this initiative throughout local networks including community-based sites, healthcare facilities and public health institutions

VCT is considered as one of the most cost-effective public health interventions for screening, and through the pre- and post- counseling sessions, it provides an opportunity to understand the personal risk of infections, encourage healthy behaviors, increase awareness of personal serostatus, prevent transmission, improve access to medical care when needed and fight discrimination [4].

Some investigations have revealed limited and highly variable levels of HIV knowledge across different population groups with diverse socio-demographic characteristics. Many of these studies in Italy have focused on the university population, highlighting outcomes of restricted awareness, even among students in healthcare-related fields [5,6,7,8].

Evaluating the general population’s knowledge and awareness of HIV and HCV is crucial in developing targeted and effective strategies to address these epidemics.

This knowledge can help tailor interventions to different population strata, such as students and adults, and increase the impact of prevention and care efforts. Here, we report the results of an individual questionnaire regarding HIV and HCV awareness and knowledge, which was offered to participants during the VCT session. In particular, the study aimed to compare knowledge and attitudes towards HIV and HCV between different subgroups such as age groups (18–40 vs. >40) and sexual orientations (heterosexual vs. LGBTQ+).

## 2. Methods

### 2.1. Study Design

This cross-sectional study was based on a self-administered questionnaire and conducted during the European Testing Week in May 2022 and World AIDS Day on 1 December 2022.

In 2022, the Infectious and Tropical Diseases Department of the ASST Spedali Civili of Brescia and the University of Brescia, together with Brescia Municipality, non-governmental organizations, and civil societies, set four days dedicated to offer voluntary counseling sessions and a rapid anonymous screening test for HIV and HCV to the general population in Brescia. Specialized health staff (infectious disease medical doctors and nurses) and a mobile unit reached the designated points of the city to offer the promotion and screening service.

### 2.2. Participants and Settings Population

Brescia is one of the major cities in the Lombardy region, in Northern Italy. In 2021, the population of Brescia comprised 197,304 inhabitants, out of which 101,854 (61.5%) were females. The majority (119,733; 60.7%) were aged over 40 years, while 49,639 (25%) were between 18 and 40 years of age [9].

The focus of this study was on the adult general population residing within the city of Brescia. To achieve this, a collaborative effort was undertaken with officials from the Municipality of Brescia to identify and select representative areas for sampling. These areas included the city center, located at the main square (referred to as C), the vicinity around the university accommodating students (referred to as A), and two peripheral zones with substantial foot traffic attributed to the presence of numerous businesses, factories, and supermarkets. These peripheral zones (referred to as B) attracted a diverse population, including residents, employees, and individuals facing socio-economic difficulties.

### 2.3. Questionnaire

An anonymous self-administered multiple-choice paper-pencil questionnaire was offered to everyone who performed the screening test or requested a counseling session (Table 1). We offered 333 people the opportunity to complete the questionnaire before taking the screening test and counseling sessions.

The questionnaire included 19 questions: four regarding the demographic characteristics such as biological sex, age, education, and sexual orientation (Q1, Q2, Q3, Q4), 11 regarding the knowledge, attitudes towards, and perception of HIV (Q5, Q6, Q7, Q8, Q9, Q10, Q11, Q12, Q13, Q14, Q15) and four questions about knowledge, attitudes towards, and perception of HCV (Q16, Q17, Q18, Q19).

### 2.4. Analysis

A descriptive analysis was performed on the demographic characteristics of the population and on the knowledge and attitudes with respect to the two diseases covered by the questionnaire.

Categorical variables are expressed as percentages and analyzed via contingency tables followed by Fisher’s exact test. All analyses were performed using GraphPad Prism 9.0 statistical package.

In order to obtain a more detailed picture of the respondents, we evaluated two categorized variables: age and sexual orientation. Consequently, results were analyzed for four different groups of respondents: people aged 18–40; people over 40 years old; heterosexual and LGBTQI+ (lesbian, gay, bisexual, transgender, queer).

We conducted an analysis to assess the level of knowledge in different demographics with regard to HIV and HCV together. We included four common questions related to both the diseases, regarding the mode of transmission (Q8, Q16), vaccines (Q11, Q17), available therapies (Q10, Q18), and testing (Q5, Q19). Based on the respondents’ answers, we categorized their knowledge levels as either high or low for both viruses. Specifically, people who answered three or four of the four questions correctly regarding each of the two infections were classified as having a high level of knowledge, and those who answered two or fewer questions correctly for each virus were classified as having a low level of knowledge. Additionally, we identified a subgroup of participants with “uneven knowledge”, those who had discordant knowledge levels between HIV and HCV.

Conclusively, a logistic regression analysis was performed to ascertain the factors linked to the high knowledge levels (people who answered three or four of the four questions correctly for each virus) pertaining to both HIV and HCV. The independent variables considered in this assessment encompassed Sex, Sexual Orientation, Age, and Education Level. Categorical variables are expressed as percentages and analyzed by contingency tables followed by Fisher’s exact test with 95% Confidence Interval (CI). All analyses were performed using GraphPad Prism 9.0 statistical package.

## 3. Results

Of all people that accepted and submitted the completed questionnaire, 123 (37%) were from area-A, 105 (31.5%) were from Area-B, and 105 (31.5%) were from Area-C. Overall, gender was balanced, with 171 (51%) females. About half of the respondents (n = 170; 51%) were aged between 18 and 25 years old, while 29 (8.7%) were aged over 60 years old.

More than half of the respondents (n = 207; 62%) were university students or had graduated from university, while 92 (27.6%) had a high school diploma or were still attending high school. Among people aged < 40 years old, 257 (n = 247; 96.1%) had higher educational levels as high school students or high school diploma and university students, or graduates. Regarding sexual orientation, the vast majority of respondents (n = 264; 79%) stated that they were heterosexual, 63 (18.9%) homosexual, bisexual, fluid, or another sexual orientation, and 6 (1.8%) respondents preferred not to declare it. All the population characteristics are shown in Table 2.

### 3.1. HIV Knowledge and Attitudes

More than half of the respondents (55%; 183) had never had an HIV test. Overall, 170 (51%) subjects knew where to access it if necessary; of these, 38 (60%) were identified as LGBTQ+ people (those identified themselves as homosexual, bisexual, fluid, other) and 130 (49.2%) were heterosexuals.

Overall, only 107 (32%) of respondents knew how HIV is transmitted (Q8). Major differences were shown between different age groups, where people under the age of 40 had a significantly higher correct response rate than people over 40 (n = 101; 39% versus n = 6; 7.8%, *p* < 0.00001). Similarly, almost half of LGBTQI+ people (n = 28; 44.4%) gave the correct answer, versus 30% (n = 79) of heterosexuals (*p* = 0.0359). In particular, over half (n = 188; 56.5%) of the respondents were aware that the risk of transmitting HIV to a newborn by a pregnant woman adhering to therapy is less than 1% (Q9). Here, again, people under 40 years showed a significantly higher response rate versus people over 40 years (n = 157; 61% vs. n = 31; 40%; *p* = 0.0023), as well as LGBTQI+ people who had a higher response rate versus heterosexuals (n = 40; 63.5% vs. n = 146; 55%, NS). Moreover, only 15 (19.7%) of those aged over 40 were aware that undetectable = untransmitable (Q13), while 123 (48%) of people under 40 years gave the correct answer (*p* < 0.00001). Significant differences were also shown between LGBTQ+ and heterosexuals (n = 34; 54% and n = 104; 39%, respectively, *p* = 0.0464).

Overall, 268 (80.5%) of respondents knew that people living with HIV who regularly take antiretroviral therapy have a similar life expectancy to people living without HIV(Q10). While 218 (85%) people under 40 knew about it, only 50 (66%) of those over 40 gave the correct answer (*p* = 0.0137). Both heterosexual and LGBTQ+ people showed a similar level of knowledge (n = 217; 82% and n = 49; 78%, respectively, NS).

Two thirds of respondents (n = 225; 67.5%) knew that there is still no vaccine that can prevent HIV infection (Q11). People over 40 are the group that showed the biggest gaps on this issue, where only 36 (47%) gave the correct answer, versus 189 (74%) of people under 40 (*p* < 0.001).

Only 75 (22.5%) of respondents had ever heard about PrEP (pre-exposure prophylaxis) (Q12), with LGBTQ+ people showed better awareness than heterosexual individual (n = 27; 43% versus n = 48; 18%, *p* < 0.0001), and with those under 40 exhibited greater knowledge than their older counterparts (26.5%, n = 68 vs. 9.2%, n = 7, respectively, *p* = 0.001).

About half of respondents (57.6%; 192) said they would cohabit with a person living with HIV (Q15). The major rejection has been found among those over 40 (n = 27, 35.5% vs. n = 165, 64% in people under 40, *p* < 0.001).

**Figure 1:**
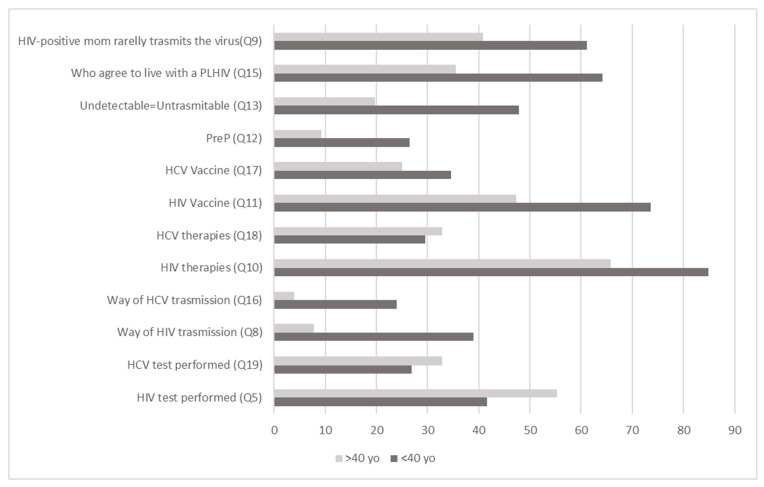
Percentages (%) of correct answers between age groups. Q5, Q19, Q8, Q16, Q10, Q18, Q11, Q17, Q12, Q13, Q15, Q9: questions 5, 19, 8, 16, 10, 18, 11, 17, 12, 13, 15, 9 of the questionnaire. <40: age group under 40 years old; >40: age group over 40 years old; PLHIV: people living with HIV.

**Figure 2:**
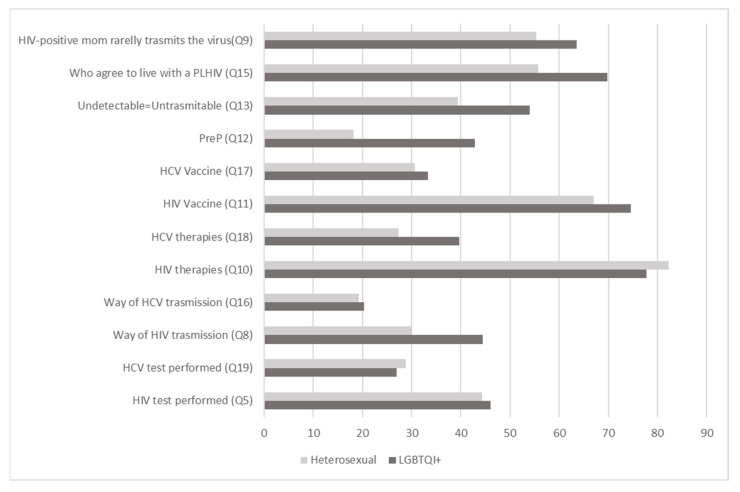
Percentages (%) of correct answers between sexual orientation groups. Q5, Q19, Q8, Q16, Q10, Q18, Q11, Q17, Q12, Q13, Q15, Q9: questions 5, 19, 8, 16, 10, 18, 11, 17, 12, 13, 15, 9 of the questionnaire. PLHIV: people living with HIV.

Percentages of correct answers are shown in Figure 1 and Figure 2

### 3.2. Hepatitis C Virus Knowledge

More than half of the respondents (187; 56%) had never had an HCV test (Q19). No major differences could be observed between populations, where only 25 (33%) and 69 (27%) of the over and under 40 population, respectively, responded that they had ever been tested for HCV.

Overall, only 66 (20%) of respondents knew how HCV is transmitted (Q16). No major differences were shown between LGBTQ+ and heterosexuals, where 15 (23.3%) and 51 (19%) gave the correct answer, respectively (NS). People under 40 had a higher correct response rate than older individuals (n = 62; 24% versus n = 3; 3.9%, *p* = 0.000).

Regarding knowledge of the HCV vaccine, the trend was similar in all the groups. Overall, 32.4% (n = 108) of the subjects knew that there is still no vaccine that can prevent HCV infection (Q17). The participants responded similarly regarding the current antiviral therapies (Q18), and 29.4% (n = 98) were aware that it is possible to heal the HCV infection by eliminating the virus. Percentages of correct answers are shown in Figure 1 and Figure 2.

### 3.3. Comparing HIV and HCV Knowledge

Overall, by comparing the responses to the same four questions for HIV and HCV (Q5, Q8, Q10, Q11 and Q19, Q16, Q17, Q18), we observed a higher proportion of individuals who achieved 100% scores (high knowledge level) on HIV questions compared to HCV (n = 56 out of 330; 16.8% vs. n = 11 out of 330; 3.3%). Additionally, for those who scored 75% (medium knowledge level), there was a significant difference, with 27% (90 out of 330) and 7.8% (26 out of 330) correct answers for HIV and HCV, respectively.

**Figure 3:**
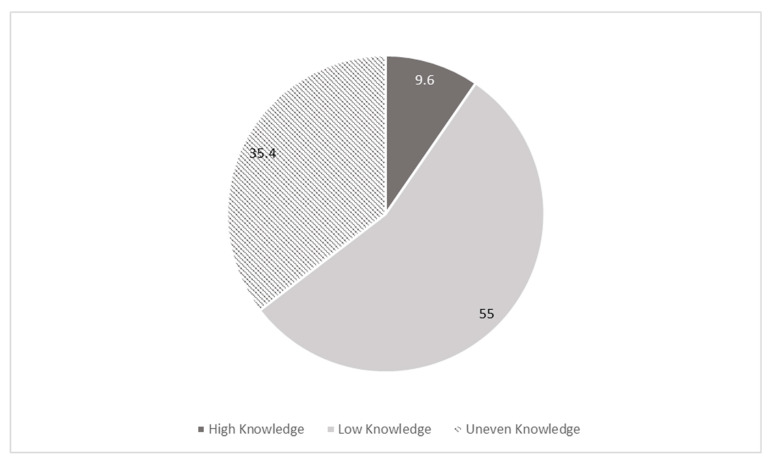
Distribution of knowledge of HIV and HCV. Percentage (%). High knowledge: those who achieved 3 or 4 (out of 4) regarding both the viruses. Low knowledge: those who achieved 0, 1, or 2 (out of 4) regarding both the viruses. Uneven knowledge: who achieved inconsistent knowledge of HIV and HCV. Evaluated questions: (Q5, Q8, Q10, Q11 and Q19, Q16, Q17, Q18).

Evaluating the knowledge of both infections together, It can be observed that there is a wide discrepancy between the subgroup with the score of correct answers between high knowledge and the people with low knowledge. In particular, people with high scores for HIV and HCV together represent only 9.6% (32 of 333) of the overall population (Figure 3).

We analyzed the sub-population according to their level of knowledge of both HIV and HCV. We found that 14.6% (25 of 171) of women and 4.4% (7 of 159) of men had high levels of knowledge. Regarding age, we found that 12.2% (31 of 257) of people under 40 had high knowledge levels compared to older respondents, with only 1.3% (1 of 76). Regarding educational level, those with higher education levels had greater knowledge (13.5%, 28 out of 207) compared to the group with low education levels—3.2% (4 out of 124).

**Figure 4:**
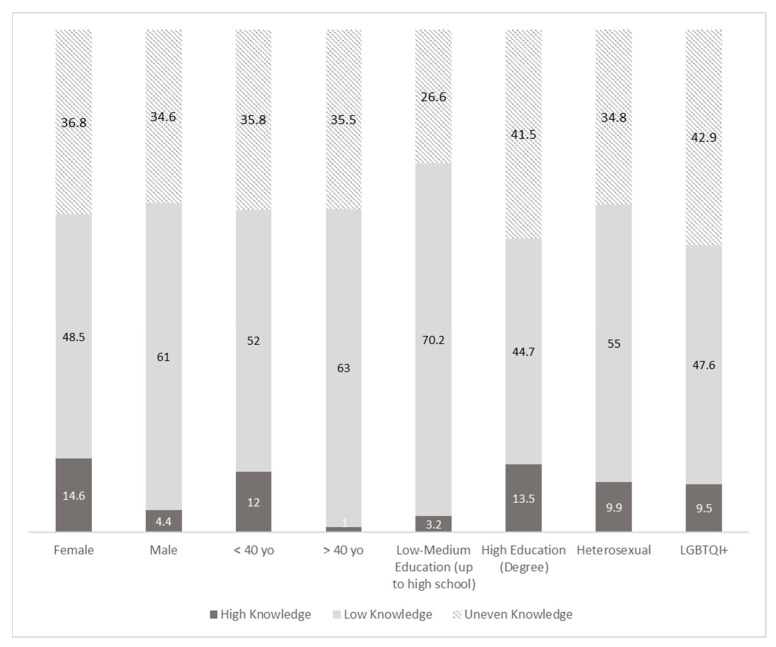
Distribution of knowledge levels of HIV and HCV together. Percentage (%). High levels of knowledge: who achieved 3 or 4 (out of 4) in both the virus. Low knowledge: who achieved 0, 1 or 2 (out of 4) regarding both viruses. Uneven knowledge: who achieved inconsistent knowledge of HIV and HCV. F: female, M: male; <40: under 40, >40: over 40.

Additionally, evaluating low knowledge levels, 48.5% (83 of 171) of women and 61.0% (97 of 159) of men had low levels of knowledge of both viruses. Regarding age, 52.1% (134 of 257) of people under 40 had high knowledge levels compared with the older group with only 63.2% (48 of 76). Considering educational level, the group with high education had a lower prevalence of low knowledge (44.7% 93 out of 207) compared to those with low-medium education levels—70.2% (87 out of 124) (Figure 4).

Ultimately, we conducted a logistic regression analysis to investigate the factors linked to elevated levels of HIV and HCV knowledge. Higher education, encompassing both individuals holding university degrees and those enrolled as college students, emerges as the variable correlated with increased of knowledge of HIV (OR 5.30, *p* = 0.003, 95% CI 1.77–15.89), but not of HCV (Table 3).

## 4. Discussion

Public awareness of infectious diseases plays an essential role in disease control, as shown during the COVID-19 pandemic [10], and the scarce or lack of knowledge of infectious diseases leads to low detection rates, treatment interruptions, discrimination, and stigma. Furthermore, a high level of knowledge of infectious diseases could not only help the general population protect themselves but also promote screening in at-risk groups, encourage suspected infected patients to seek early medical care and more comprehensive care. We conducted this survey to provide an overall picture in our city of the public’s awareness of infectious diseases that the WHO have planned to end as epidemics by 2030—HIV and HCV.

The main results of our study reflect that, in general, almost 70% of those interviewed had null or very low levels of knowledge of HCV and almost 30% of HIV infection, with important differences pertaining to age, sexual orientation, and education. The results from the logistic regression analysis concerning knowledge levels reveal that respondents with higher education backgrounds (including university students and graduates) displayed a higher likelihood of providing accurate answers to the HIV-related questions, according to a previous study [5]. In addition, just under half of the participants had ever been tested for HIV and/or HCV. In Naples, south Italy, a small study (244 participants attending a HIV counseling and testing service) was conducted before 2010 about knowledge and attitudes regarding HIV infection. Only 25% of responders correctly identified HIV modes of transmission, and only 21% had received previous HIV testing [5]. Our study shows that after more than 10 years there are still huge gaps in this topic in Italy.

The level of knowledge regarding the modes of HIV transmission appeared to be quite variable in our overall study population, with better results in the younger and LGBTQI+ groups. Similar results were found in studies conducted in Jordan, China, and Saudi Arabia [11,12,13]. This group also appears to be more aware of the efficacy of HIV drug therapy and life expectancy for the PLWH.

Notwithstanding, our study reveals an uneven level of awareness regarding the notion that undetectable = untransmittable, with greater knowledge observed among the LGBTQI+ and younger age cohorts. A recent Italian survey of university students revealed that only 3 out of 10 of those interviewed were aware that in Italy, new cases of HIV infection are mainly attributable to heterosexuals [8]. The LGBTQI+ usually have more awareness of HIV infection, and the incorrect notion that HIV is a health problem mainly affecting this group could generate the mistaken self-perception of a low risk of HIV infection among heterosexual people.

Regarding PrEP, we found a poor level of knowledge in all the considered groups, with slightly better scores in the LGBTQI+ group. The less informed subgroup appeared to be the older group. These data are consistent with findings in from the United States [14] and in a student cohort study in Italy [15]. Preventive measures including health information and preventive strategies must be implemented in our area.

Poor HIV-related knowledge has been closely linked with biased attitudes [12,16]. Similarly, we observed a persistent prevalence of stigma mainly among the older population, as evidenced by the reluctance to cohabit with individuals living with HIV. Again, here we showed how people over the age of 40 are influenced by outdated information, when HIV was a highly stigmatized and deadly disease.

In a former European study, greater knowledge of HIV infection was linked to younger age groups and higher levels of education, implying that educational initiatives could positively influence the level of knowledge regarding these diseases [17]. Our results suggest the need to implement health educational campaigns for the older population with targeted and effective strategies tailored to different population strata, with information on the mode of transmission of the disease and to develop a series of policies and measures to fight against HIV stigma. This is also relevant if we consider that in Italy, the median age of HIV diagnosis is 41, with an increasing prevalence of late diagnosis [18].

The level of knowledge of HCV has previously been evaluated in various countries with inconsistent results. Surveys in Ethiopia (Healthcare workers of a University Medical Center), and Egypt (clinically diagnosed HCV patients) showed a medium-high level knowledge of HCV infection (60.9% and 49%, respectively) [19,20]. In Saudi Arabia, a country with 1.1% HCV prevalence, 53% of the participants of one survey (general population) had a low level of knowledge with a low HCV testing rate (17.8%) [21]. In our study, only 11% of participants had medium/high level of HCV knowledge

Another important result is obtained by comparing the knowledge of HIV and HCV. A previous study among health workers in Malawi showed that knowledge of HCV was lower when compared with HIV/AIDS [22]. In our study, knowledge of HIV is also greater than knowledge of HCV, although HIV prevalence is estimated to be lower [23]. In particular, the population with medium-high scores was mostly women, aged below 40 years, and almost all were university students or graduates. On the other hand, regarding the broader population who had low or insufficient knowledge, the population was equally distributed between males and females; in this group, 70% were under the age of 40, while half were university students or graduates.

Several studies have reported inadequate knowledge regarding HCV transmission. This phenomenon may be partially explained by the lower prevalence of HCV infection in certain countries [24,25], which could not be applicable in Italy, where 1–1.5% of the population is estimated to be affected by HCV [26]. This knowledge gap may also be present among high-risk populations, such as older people, who would benefit the most from educational and preventative interventions. The large number of undiagnosed HCV cases is the biggest concern as risk factor, and screening is suboptimal.

For this reason, the Lombardy region promoted a population screening for HCV infection, and specifically offered it to people born from 1969 to 1989 [26].

The main limitations of this study include a limited number of interviews and bias related to the self-reported nature. Another limitation pertains to the presence of a selection bias among individuals who voluntarily opt to participate in the screening process. Those who proactively utilize the mobile van units are likely to possess a heightened perception of personal risk compared to their non-participating counterparts. This potential bias could introduce systematic variations in the study cohort, potentially leading to an overrepresentation of individuals who self-identify as being at risk.

## 5. Conclusions

This study demonstrated that the current level of awareness of these main infectious diseases in citizens residing in a large, mainly industrial city in northern Italy, is still very low and further effective health education campaigns for major infectious diseases are urgently needed. Health information should focus on preparedness, confidence, counseling, and prevention strategies, thereby enhancing the public’s sense of social responsibility (less than 45% of the subjects had ever been tested for HIV and 44% for HCV). Higher education level and younger age play important and positive roles in the knowledge of these infectious diseases [27]. Education is important in forming attitudes related to health-changing behaviors and awareness together with specific health campaigns such as those that the WHO promotes around the world [28].

Our findings may serve as a roadmap for education programs to prevent and to facilitate early diagnosis and treatment of these infections and to eliminate stigma, while no vaccines are available for HIV and HCV. Clearly, much more work is needed to bring these diseases of public health importance to the attention of our population and authorities, especially those in certain subgroups. Our study highlights that misconceptions about HIV and HCV should be addressed in prevention and education programs, whose target should also be specific populations, such as older people that are less frequently involved in the efforts to address these epidemics. This awareness should result in network building and collective actions that benefit all, contributing expertise, resources, knowledge, and experience to achieving a common public good.

## Figures and Tables

**Table 1:** Self-Administered Multiple-Choice Questionnaire.

Biological Sex Q1	F	M	Other			
Age Q2	18–25	25–30	30–40	40–50	50–60	>60
Educational level Q3	Primary School Diploma	Middle School Diploma	High School Diploma	High School Student	University Student	University Diploma
Sexual Orientation Q4	Heterosexual	Homosexual	Bisexual	Fluid	Other	
HIV						
Have you ever performed an HIV test before? Q5	Yes	No				
Do you know where to perform the HIV test in your city? Q6	Yes	No				
Do you know people living with HIV? Q7	Yes, my partner	Yes, a relative	No			
How HIV can be transmitted Q8 * more than one correct answer	Sharing cutlery	Mosquito bites	Infected blood	Saliva	Unprotected sex	Pregnancy
A mom living with HIV, regularly taking antiretroviral therapy can transmit the HIV virus during pregnancy: Q9	Yes, in 90% of cases	Yes, in 50% of cases	In less than 1% of cases			
With the current drug therapies, People living with HIV could have a quality of life similar to HIV-negative people Q10	Yes	No	I do not know			
Do you think there is a vaccine against HIV? Q11	Yes	No	I do not know			
Do you know what’s the PreP? Q12	Yes	No	I do not know			
People living with HIV, regularly taking antiretroviral therapy and having “viremia 0” can transmit the HIV virus Q13	Yes	No	I do not know			
Do you know the meaning of “Undetectable”? Q14	Yes	No	I do not know			
Do you agree to live with People living with HIV? Q15	Yes	No	I do not know			
HCV						
HCV virus can be transmitted through * more than one correct answer Q16	Mosquito bites	By air (cough or sneeze)	Unprotected sex	Infected blood	During the pregnancy	
There is a vaccine that prevents HCV Q17	Yes	No	I do not know			
The current therapies are able to treat the infection and eliminate the hepatitis virus HCV Q18	Yes	No	I do not know			
Have you ever performed an HCV test before? Q19	Yes	No	I do not know			

Q1, Q2, Q3, Q4, Q5, Q6, Q7, Q8, Q9, Q10, Q11, Q12, Q13, Q14, Q15, Q16, Q17, Q18, Q19: questions 1, 2, 3, 4, 5, 6, 7, 8, 9, 10, 11, 12, 13, 14, 15, 16, 17, 18, 19 of the questionnaire. * more than one correct answer for Q8 and Q16.

**Table 2:** Demographic characteristics. n = number, % = percentage.

		Overall		<40		>40		Heterosexual		LGBTQ+	
		n	%	n	%	n	%	n	%	n	%
	TOT	333	100.0	257	77.2	76	22.8	264	79.3	63	18.9
Biological **Sex Q1**	F	171	51.4	145	43.5	26	7.8	142	42.6	28	8.4
	M	159	47.7	111	33.3	48	14.4	120	36.0	34	10.2
	other	3	0.9	1	0.3	2	0.6	2	0.6	1	0.3
**Age Q2**	18–25	170	51.1	170	51.0	-	-	136	40.8	34	10.2
	25–30	57	17.1	57	17.1	-	-	43	12.9	13	3.9
	30–40	30	9.0	30	9.0	-	-	21	6.3	8	2.4
	40–50	19	5.7	-	-	19	5.7	17	5.1	0	0.0
	50–60	28	8.4	-	-	28	8.4	23	6.9	5	1.5
	>60	29	8.7	-	-	29	8.7	24	7.2	3	0.9
**Educational level Q3**	Primary School Diploma	5	1.5	1	0.3	4	1.2	2	0.6	1	0.3
	Middle School Diploma	27	8.1	7	2.1	20	6.0	22	6.6	3	0.9
	High School Diploma	58	17.4	32	9.6	26	7.8	51	15.3	7	2.1
	High School Student	34	10.2	28	8.4	6	1.8	22	6.6	11	3.3
	University Student	139	41.7	137	41.1	2	0.6	112	33.6	27	8.1
	University Diploma	68	20.4	50	15.0	18	5.4	53	15.9	14	4.2
	NA	2	0.6	2	0.6	0	0.0	2	0.6	0	0.0
**Sexual Orientation Q**	Heterosexual	264	79.3	200	60.1	64	19.2	264	79.3	-	-
	Homosexual	18	5.4	16	4.8	2	0.6	-	-	18	5.4
	Bisexual	35	10.5	32	9.6	3	0.9	-	-	35	10.5
	Fluid	4	1.2	4	1.2	0	0.0	-	-	4	1.2
	Other	6	1.8	3	0.9	3	0.9	-	-	6	1.8
	NA	6	1.8	2	0.6	4	1.2	6	1.8	-	-

Q1, Q2, Q3, Q4: questions 1, 2, 3, 4 of the questionnaire. <40: age group under 40 years old; >40: age group over 40 years old. LGBTQ+: homosexual, bisexual, fluid, other. NA refused to answer.

**Table 3:** Multivariate logist regression model results.

	High Knowledge of HIV			High Knowledge of HCV		
Log likelihood = −213.05	Odds Ratio	*p*-Value	95% CI	Odds Ratio	*p*-Value	95% CI
**Years of age—**Ref. Category: < 40						
> 40	1.10	0.760	0.58–2.09	0.63	0.452	0.19–2.07
**Sex—**Ref. Category: Female						
Male	0.71	0.162	0.44–1.14	0.52	0.099	0.24–1.13
**Sexual Orientation—**Ref. Category: Heterosexual						
LGBTQ+	1.19	0.562	0.65–2.17	1.10	0.825	0.44–2.74
**Education—**Ref. Category: Primary and Middle school diploma						
High School students and diploma	2.38	0.119	0.79–7.12	1.72	0.640	0.17–17.16
University students and diploma	5.30	0.003 *	1.77–15.89	54.44	0.189	1.48–41.11

* Significant at *p* < 0.005. >40: age group over 40. LGBTQ+: homosexual, bisexual, fluid, other. High school: high school diploma and high school students; University: university students and university diploma.

## Data Availability

Data are contained within this article.
